# A Wheat WRKY Transcription Factor TaWRKY46 Enhances Tolerance to Osmotic Stress in transgenic Arabidopsis Plants

**DOI:** 10.3390/ijms21041321

**Published:** 2020-02-15

**Authors:** Xiaorui Li, Yan Tang, Chunju Zhou, Lixin Zhang, Jinyin Lv

**Affiliations:** College of Life Sciences, Northwest A&F University, Yangling 712100, China; lxr0515@126.com (X.L.); tangyanyan418@163.com (Y.T.); zhchju@nwsuaf.edu.cn (C.Z.); zhanglixin@nwsuaf.edu.cn (L.Z.)

**Keywords:** wheat, transcription factor, TaWRKY46, bioinformatics, osmotic stress, mannitol

## Abstract

WRKY transcription factors play central roles in developmental processes and stress responses of wheat. Most WRKY proteins of the same group (Group III) have a similar function in abiotic stress responses in plants. *TaWRKY46*, a member of Group III, was up-regulated by PEG treatment. TaWRKY46-GFP fusion proteins localize to the nucleus in wheat mesophyll protoplasts. Overexpression of *TaWRKY46* enhanced osmotic stress tolerance in transgenic *Arabidopsis thaliana* plants, which was mainly demonstrated by transgenic *Arabidopsis* plants forming higher germination rate and longer root length on 1/2 Murashige and Skoog (MS) medium containing mannitol. Furthermore, the expression of several stress-related genes (*P5CS1*, *RD29B, DREB2A, ABF3, CBF2,* and *CBF3*) was significantly increased in *TaWRKY46-*overexpressing transgenic *Arabidopsis* plants after mannitol treatment. Taken together, these findings proposed that TaWRKY46 possesses vital functions in improving drought tolerance through ABA-dependent and ABA-independent pathways when plants are exposed to adverse osmotic conditions. *TaWRKY46* can be taken as a candidate gene for transgenic breeding against osmotic stress in wheat. It can further complement and improve the information of the WRKY family members of Group III.

## 1. Introduction

Drought is one of the most hazardous environmental abiotic stressors, which adversely influences the growth and development of wheat (*Triticum aestivum* L.) [1,2]. It has caused an average of 13.7% loss in cereal production worldwide over the past few decades [3]. In Northwest China, wheat is cultivated under rain-fed conditions and encounters severe water shortage because of the low rainfall [4]. Uncovering the sophisticated underlying mechanisms that allow wheat to adapt to various detrimental stresses has vital significance for the improvement of wheat drought tolerance [5,6]. Several transcription factor (TF) families, such as WRKY, MYB, and NAC, have been reported to play key roles in response to drought stress in plants.

WRKY TFs mediate signal transduction pathways by regulating the transcription of target DNA and eventually leading to stress tolerance in plants [7,8,9,10,11]. The WRKY family is characterized by the WRKY domain, which comprises a short conserved sequence WRKYGQK at the N-terminal and a zinc finger motif at the C-terminal [12,13]. According to the number of WRKY domains and zinc-finger structures, the WRKY family members are divided into three groups. Two WRKY domains and a C_2_H_2_ (CX_4-5_CX_22-23_HXH) zinc finger exist in Group I. Group II a-e and Group III contain one WRKY domain and a C_2_H_2_ motif or a C_2_HC (CX_7_CX_23_HXC) motif, respectively [14]. The WRKY domain controls transcription by specifically binding to the upstream W-box sequence of the target genes [15,16].

As a hexaploid plant, the wheat genome consists of the A, B, and D genomes with 15,966 Mb in size [17,18]. In a previous study, 171 WRKY TFs were identified in wheat [19]. Less than one-third of wheat *TaWRKYs* have been functionally analyzed so far. Recent studies have emphasized the involvement of TaWRKY TFs in various biotic and abiotic stress responses and plant growth and development processes, such as pathogen defense [20], high salt stress [21,22], extreme temperature [23,24], drought stress [21,22,25,26] and senescence [15].

The WRKY members of Group III have been identified as participants in abiotic stress responses in plants. *AtWRKY46* played roles in regulating plant responses to osmotic stress and stomatal movement [27]. *GhWRKY33* functioned as a negative regulator in drought stress response and participates in the ABA signaling pathway [28]. The overexpression of *VlWRKY48* enhanced plant drought tolerance by multiple mechanisms. Additionally, *VlWRKY48* controlled the resistance to powdery mildew infection [29]. The function of the WRKY members that belonged to Group III is conserved. Whether or not the wheat WRKY members of Group III play a role in abiotic stress responses is still unclear.

In our previous work, 45 WRKY TFs which belonged to Group III were identified in wheat [19]. To assess the function of wheat Group III members, a Group III gene *TaWRKY46* (TRAES3BF051200110CFD_t1) is cloned in this study. Our results showed that overexpression of *TaWRKY46* promoted the germination rate and boosted the root growth in *TaWRKY46*-overexpressing *Arabidopsis* plants under osmotic stress. Overexpression of *TaWRKY46* also induced stress-related genes in transgenic *Arabidopsis* plants, which suggested that TaWRKY46 played an important role in enhancing osmotic tolerance through ABA-dependent and ABA-independent pathways. This study provides more information for further investigation into the underlying mechanisms of *TaWRKY* genes that are involved in osmotic-related regulatory networks.

## 2. Results

### 2.1. Identification of TaWRKY46 and its Transcription Profiles under Various Stress Conditions

**Figure 1:**
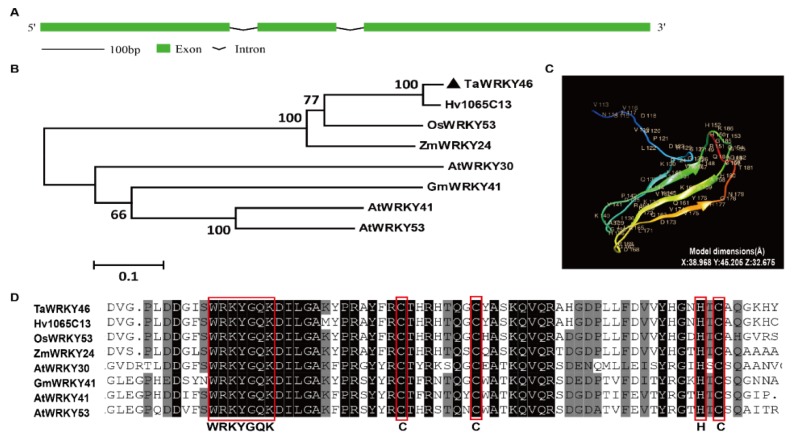
Sequence analysis of TaWRKY46. (**A**) A schematic representation showing the gene structure of TaWRKY46. (**B**) Phylogenetic relationship of TaWRKY46 with its orthologous in other plant species. The accession numbers of the *WRKYs* are listed in Supplementary Table S1. (**C**) Tertiary structure of TaWRKY46 protein. A total of 71 amino acid residues were modelled with 100% confidence by the single highest scoring template. Image was colored by rainbow from N to C terminus. The number and capital letter represent the order and abbreviation of amino acids. (**D**) Sequence alignment of TaWRKY46 and its homologs. Black and grey background represent 100 and 75% similarity, respectively. The completed WRKY domain and C_2_HC zinc finger motif are circled in red boxes.

*TaWRKY*46 comprised the splicing of three exons and two introns. The length of wheat *WRKY*46 was 1062 base pairs (bp). The sizes of the introns were 83 bp and 94 bp, respectively (Figure 1A). TaWRKY46 and its orthologous from other plant species were used to construct a phylogenetic tree. The result showed that TaWRKY46 had the closest relationship with Hv1065C13 (AK357930.1) from *Hordeum vulgare* due to its high identity (Figure 1B). The TaWRKY46 protein had 294 amino acid residues. A total of 71 amino acid residues (24% of sequence) had been modelled with 100% confidence by the single highest scoring template. The image of the tertiary structure of TaWRKY46 was colored by rainbow from N to C terminus. It had four β-sheets which were displayed in the tertiary structure. The model dimensions of tertiary structures were also given in detail (Figure 1C). Multiple sequence alignment revealed that all WRKYs had the conserved WRKYGQK motif and amino acid residues. TaWRKY46 was assigned to Group III, due to the existence of one WRKY domain and a C_2_HC zinc finger motif (Figure 1D).

**Figure 2:**
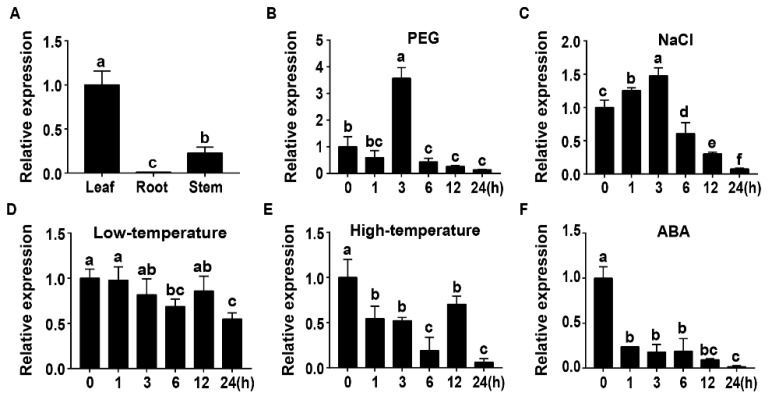
Transcription profiles of *TaWRKY46*. (**A**) Organ expression assay of *TaWRKY46* in different wheat organs (leaf, root, and stem). Transcription profiles of *TaWRKY46* under 20% PEG 6000 (**B**); 200 mM NaCl (**C**); 4 °C (**D**); 42 °C (**E**); 100 μM ABA (**F**) treatments in wheat leaves. The transcriptional level at time point 0 (for the multiple stress experiments) and the leaf (for the organ expression assay) was defined as 1.0.

To dissect potential functions, the expression of *TaWRKY46* was investigated in different tissues and various stress conditions by qRT-PCR. The results showed that *TaWRKY46* was expressed in all organs, with the higher and lower transcriptional levels in the leaf and root, respectively (Figure 2A). Transcription of *TaWRKY46* was not affected by low-temperature, but was repressed by high-temperature and exogenous ABA treatments (Figure 2D–F). The *TaWRKY46* gene was slightly induced by NaCl at a maximum level of about 1.48-fold at 3 h (Figure 2C). *TaWRKY46* was markedly induced after PEG treatment, and increased by 3.57-fold at 3 h (Figure 2B).

### 2.2. Subcellular Location of TaWRKY46

**Figure 3:**
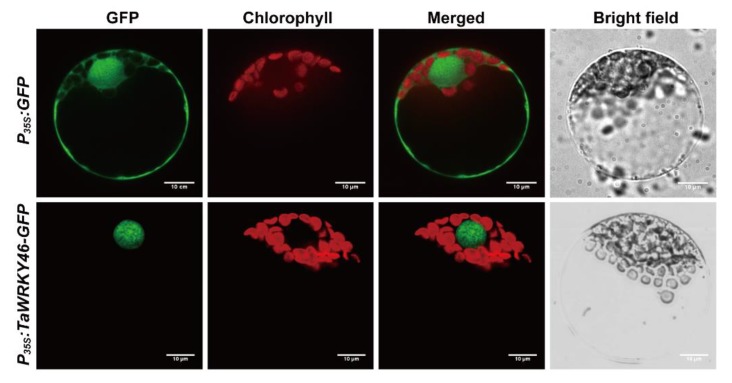
Subcellular localization of the *TaWRKY46*. *P_35S_:TaWRKY46-GFP* and *P_35S_:GFP* control vectors were transiently expressed in wheat leaf protoplasts. Scale bar = 10 μm.

The full coding region of *TaWRKY46* was cloned into the *pTF486-GFP* vector under control of the CaMV35S promoter and transformed into wheat leaf protoplasts. The empty vector was used as a control. The GFP signal of *35S:TaWRKY46-GFP* was exclusively observed in the nucleus, whereas GFP signal of the control was discovered in the cytoplasm and nucleus (Figure 3). Therefore, TaWRKY46 likely functioned as a transcription factor in the nucleus.

### 2.3. Transcriptional Activation Assay of TaWRKY46 in Yeast

**Figure 4:**
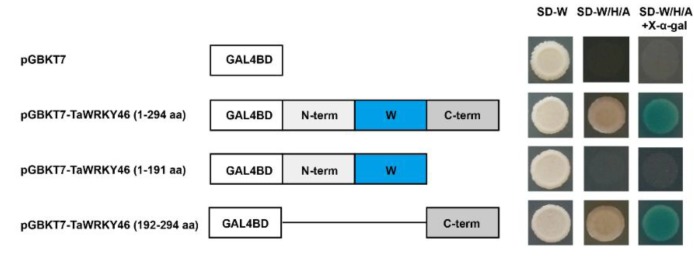
Transcriptional activity assay of TaWRKY46 protein in yeast. A schematic diagram of fused vectors exhibiting the truncated segments of TaWRKY46 that were fused to the vector *pGBKT7*. The transformants were incubated on the SD-W, SD-W/H/A, and SD-W/H/A with X-α-gal medium.

Transcriptional activation of TaWRKY46 was detected with the GAL4 yeast expression system. Yeast strain AH109 was transformed with constructions *pGBKT7-TaWRKY46* (1-294 aa), *pGBKT7-TaWRKY46* (1-191 aa), and *pGBKT7-TaWRKY46* (192-294 aa), and *pGBKT7* was taken as a negative control. The yeast cells transformed with *pGBKT7-TaWRKY46* (1-294 aa) and *pGBKT7-TaWRKY46* (192-294 aa) grew well on the SD-W/H/A medium (Figure 4). Meanwhile, yeast cells transformed with *pGBKT7-TaWRKY46* (1-191 aa) and empty vector *pGBKT7* could only survive on the SD-W medium. The yeast cells transformed with *pGBKT7-TaWRKY46* (1-294 aa) and *pGBKT7-TaWRKY46* (192-294 aa) grew well on SD-W/H/A medium, and turned blue in the presence of X-α-gal. The results demonstrated that the C-terminal region of TaWRKY46 functioned as a transcriptional activator.

### 2.4. Overexpression of TaWRKY46 Improved Osmotic Tolerance in Transgenic Arabidopsis Plants

**Figure 5:**
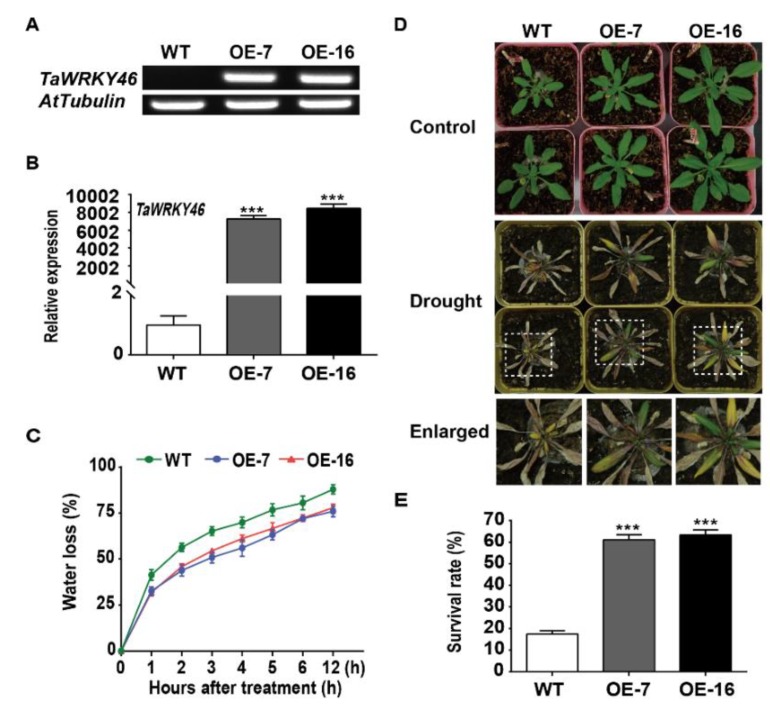
The phenotype and tolerance assay of *TaWRKY46*-overexpressing *Arabidopsis* after drought treatment. Gene validation of *TaWRKY46* in transgenic *Arabidopsis* lines by PCR (**A**) and qRT-PCR (**B**). (**C**) The water loss rate of the detached leaves. (**D**) The phenotype of *TaWRKY46*-overexpressing *Arabidopsis* plants after water withholding for 25 days. (**E**) The survival rate of *TaWRKY46*-overexpressing *Arabidopsis* plants after drought treatment. Asterisks indicate a significant difference between wild-type (WT) and transgenic *Arabidopsis* lines (*** *p* < 0.001).

The complementary DNA (cDNA) encoding TaWRKY46 was inserted into the *pBI1304* vector under the control of the CaMV35S promoter. The PCR and qRT-PCR analysis showed that *TaWRKY46* was detected in the two selected *TaWRKY46*-overexpressing *Arabidopsis* plants (OE-7 and OE-16), but not in the wild-type (WT) (Figure 5A,B). The water loss rate was evaluated at the indicated interval for both WT and *TaWRKY46*-overexpressing *Arabidopsis* plants. Compared with the WT plants, the detached leaves of *TaWRKY46*-overexpressing *Arabidopsis* plants possessed lower water loss rate (Figure 5C). The 24-day-old seedlings of WT and transgenic Arabidopsis plants were exposed to drought conditions for 25 days. All leaves of WT Arabidopsis plants were severely curled and turned yellow, while some leaves of the transgenic plants (OE-7 and OE-16) remained green (Figure 5D). After withholding water for 25 d, 17.34% of the WT plants survived while the survival rates of the transgenic Arabidopsis OE7 and OE16 lines were 61.13% and 63.33%, respectively (Figure 5E).

**Figure 6:**
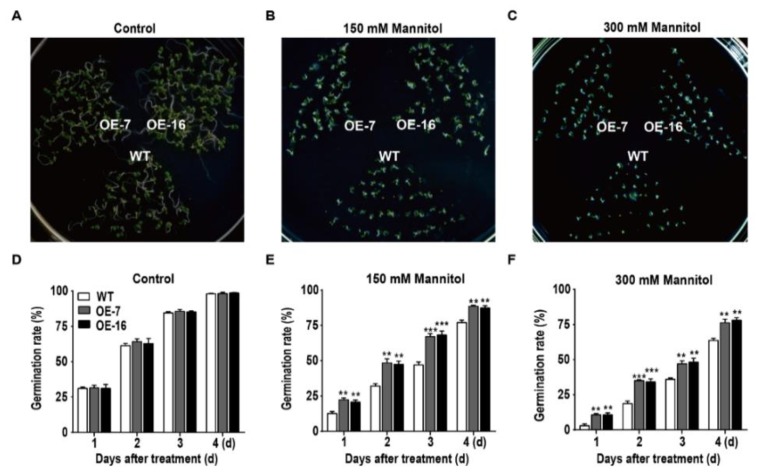
The germination rate of overexpressing *TaWRKY46* in *Arabidopsis* under osmotic stress. WT and transgenic *Arabidopsis* seeds were sown on 1/2 MS medium containing 0 mM (**A**,**D**), 150 mM (**B**,**E**), and 300 mM mannitol (**C,F**). Panels (**A**–**C**) are the photos of germination status on media after 4 days; (**D**–**F**) are the diagrams of germination rate calculated for 4 days. Asterisks indicate a significant difference between WT and transgenic *Arabidopsis* lines (**p < 0.01; ***p < 0.001).

To assess the osmotic stress tolerance of the transgenic *Arabidopsis* plants, WT and *TaWRKY46*-overexpressing *Arabidopsis* seeds were sown on 1/2 MS medium containing mannitol (0 mM, 150 mM, and 300 mM), and the seed germination rate was monitored for 4 days. Compared with WT*, TaWRKY46*-overexpressing transgenic *Arabidopsis* seeds grown on 1/2 MS medium containing mannitol showed remarkable differences in the germination rate (Figure 6A–C). More than 87.34% and 76.19% of *TaWRKY46* transgenic seeds germinated in 1/2 MS medium supplemented with 150 mM and 300 mM mannitol, respectively, compared to 77.06% and 63.72% for WT seeds (Figure 6E,F). However, the seeds planted on the 1/2 MS medium without mannitol had no obvious difference between the WT and *TaWRKY46*-overexpressing transgenic *Arabidopsis* lines (Figure 6D). The results suggested that overexpression of *TaWRKY46* enhanced osmotic tolerance during seed germination in transgenic *Arabidopsis* plants.

**Figure 7:**
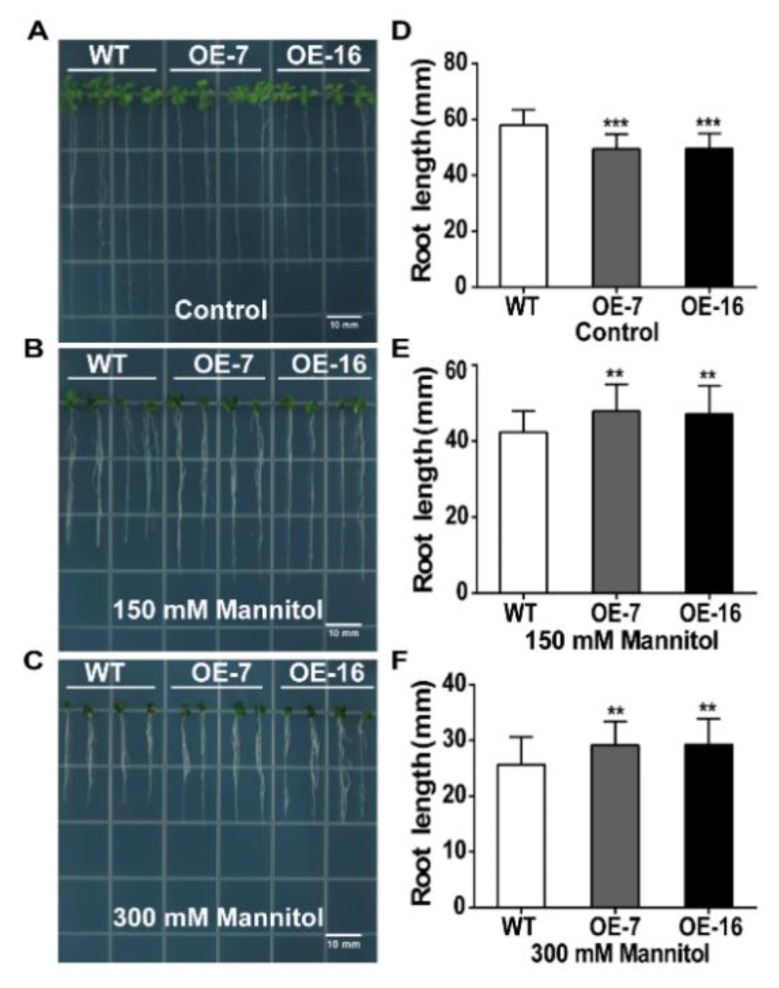
Osmotic tolerance of *TaWRKY46*-overexpressing *Arabidopsis* plants. (**A**–**C**) The phenotype of seedlings after treatments with 0 mM, 150 mM, and 300 mM mannitol for 10 days. (**D**–**F**) The root length statistics of seedlings after treatments with 0 mM,150 mM, and 300 mM mannitol for 10 days. Asterisks indicate a significant difference between WT and transgenic *Arabidopsis* lines (** *p* < 0.01; *** *p* < 0.001). Scale bar = 10 mm.

*TaWRKY46*-overexpressing *Arabidopsis* and WT seeds were grown vertically on 1/2 MS medium at 22 °C. For the mannitol treatment, 5-old-day seedlings were then grown on 1/2 MS medium supplemented with mannitol (0 mM, 150 mM, and 300 mM) in the same growth chamber for 10 days. WT plants grew a little better on 1/2 MS medium without mannitol than the *TaWRKY46*-overexpressing *Arabidopsis* plants (Figure 7A). In contrast, the overexpression lines grew noticeably better on the mannitol medium than those of the WT (Figure 7B,C). The root length results further confirmed the promoted tolerance of the overexpression of *TaWRKY46* in transgenic *Arabidopsis* plants. The root lengths of the *TaWRKY46*-overexpressing *Arabidopsis* plants were longer than those of WT plants under mannitol treatments, although mannitol stress repressed the growth of both *TaWRKY46*-overexpressing *Arabidopsis* and WT plants (Figure 7D–F). However, the root lengths of WT and *TaWRKY46*-overexpressing *Arabidopsis* plants had no differences under 100 and 200 mM NaCl treatments (Supplementary Figure S1).

### 2.5. TaWRKY46 Regulates the Expression of Stress-Related Genes Under Osmotic Stress in Arabidopsis Plants

**Figure 8:**
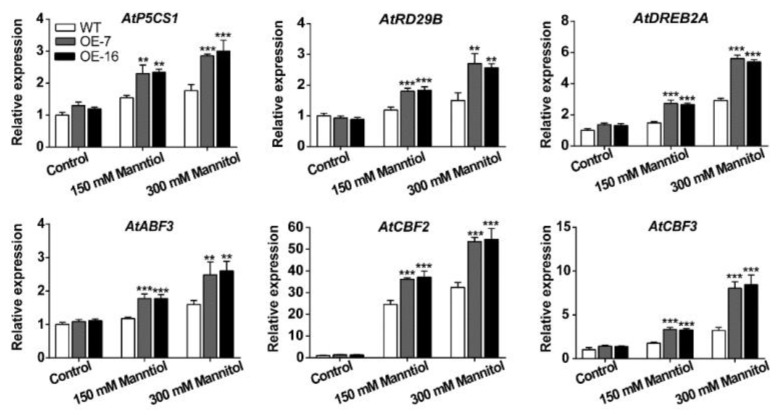
The transcriptional levels of stress-related genes in *TaWRKY46*-overexpressing *Arabidopsis* plants after 0, 150 and 300 mM mannitol treatments for 10 days. Asterisks indicate a significant difference between WT and transgenic *Arabidopsis* lines (** *p* < 0.01; *** *p* < 0.001).

The transcriptional levels of stress-related genes were detected in the 15-day-old WT and *TaWRKY46*-overexpressing transgenic *Arabidopsis* seedlings after mannitol stress. We selected six genes listed below for this experiment: Δ-1-pyrroline-5-carboxylate synthetase 1 (*P5CS1),* dehydration-responsive 29B (*RD29B*), dehydration-response element-binding protein 2A (*DREB2A*), ABA-response element (ABRE) binding factor 3 (*ABF3*), C repeat/dehydration-responsive element-binding factor 2 (*CBF2*), and *CBF3*. Compared with the WT plants, the six stress-related genes analyzed were prominently up-regulated in the *TaWRKY46*-overexpressing transgenic *Arabidopsis* lines when seedlings were exposed to 150 mM and 300 mM mannitol treatments (Figure 8). The results demonstrated that the overexpression of *TaWRKY46* enhanced osmotic tolerance by inducing the expression of some stress-related genes in transgenic *Arabidopsis* plants.

## 3. Discussion

WRKY TFs are one of the largest families of transcriptional regulators in plants, and have multiple developmental and physiological functions in response to environmental stresses [30]. In plants such as rice [31], soybean [16,32], cotton [33], *Brachypodium distachyon* [34,35], maize [36], and wheat [37], an especially large number of WRKY proteins have been identified in recent years.

In the present study, multiple sequence alignment showed that TaWRKY46 possessed the conserved WRKYGQK domain and a C_2_HC (CX_7_CX_23_HXC) motif (Figure 1D). The TaWRKY46-GFP fusion protein was exclusively localized to the nucleus of wheat protoplasts in a transient expression assay (Figure 3). It was in agreement with its putative role as a transcription factor and similar to previous reports on some other WRKY TFs [38,39]. Transcriptional activation analysis illuminated that the sequence of TaWRKY46 has transcriptional activation activity (Figure 4). It was in accordance with a senescence-associated transcription factor TaWRKY7 [15]. The transcriptional activation of truncated segments of TaWRKY46 illuminated that the C-terminal sequence of TaWRKY46 has transcriptional activation activity.

The WRKY TF members are known to be a vital section in drought stress and the ABA signal pathway [30,40]. The activation of the ABA signal transduction pathway also elevated plant drought tolerance by repressing transpiration, stomata opening, and the growth retardation factors [41]. But the expression patterns of *TaWRKY46* seemed to be inconsistent under PEG and ABA treatments (Figure 2B,F). The underlying mechanism will need further exploration.

The structural conservation of WRKY proteins determines the functional specificity in regulating gene expression. Investigations showed that most WRKY proteins of the same group have a similar function in many plant species. Gene expression of *TaWRKY46,* which belonged to Group III, was induced in the leaf under PEG treatment (Figure 2B). It implied that *TaWRKY46* might play a key role in response to osmotic stress. It was similar to the expression profiles of AtWRKY46 and AtWRKY63. AtWRKY46 plays important functions in responses to osmotic stress and stomatal movement [27]. AtWRKY63, a member of Group III, mediated responses to exogenous ABA and drought stress in *Arabidopsis* [42]. All these currently available data suggested that they may possess conserved functions in abiotic stress signaling.

The water loss rate of the detached leaves has been widely used to reflect drought tolerance in plants. The detached leaves of *TaWRKY46*-overexpressing *Arabidopsis* plants exhibited lower rates of water loss compared to the WT plants (Figure 5C). *TaWRKY46*-overexpressing *Arabidopsis* plants had the higher survival rate after drought treatment, and possessed the higher germination rate at different concentrations of mannitol (Figure 5E and Figure 6). Further phenotypic analysis showed that overexpression of *TaWRKY46* altered the length of roots in transgenic *Arabidopsis* plants under osmotic stress (Figure 7). All the results suggested that overexpression of *TaWRKY46* enhanced osmotic tolerance in transgenic *Arabidopsis* plants. Similar findings were also verified in some other WRKY transcription factors [22]. However, a remarkable difference was not observed in the root length of WT and *TaWRKY46*-overexpressing *Arabidopsis* plants after 100 mM and 200 mM NaCl treatments (Supplementary Figure S1). It was mainly because of the low induction of *TaWRKY46* gene expression under NaCl treatment.

To gain further insight into the mechanism of action of TaWRKY46 in osmotic stress at the molecular level, six stress-related genes were detected at the transcriptional level. P5CS1 is one of the core enzymes involved in proline biosynthesis. Proline is a widely distributed osmolyte that regulates the osmotic balance [43]. Herein, we found that *P5CS1* was greatly induced in transgenic *Arabidopsis* plants (Figure 8). It indicated that *AtP5CS1* was possibly regulated by TaWRKY46. There was also a possibility that the resistance ability was given by WRKY SNPs mutations [44]. *Fortunella crassifolia* WRKY40 could also specifically bind to and activate the promoter of *FcP5CS1* under salt stress [45]. The promoted expression of *P5CS1* could lead to the accumulation of osmolytes that can maintain the osmotic capacity, thus sustaining the water content.

ABA, a vital hormone, is involved in plant gene regulation networks by an ABA-dependent or ABA-independent pathway when plants combat adverse environmental stresses [5,46]. The expression of *ABF3* and *RD29B* (ABA-dependent) and *DREB2A*, *CBF2*, and *CBF3* (ABA-independent) genes were enhanced in *TaWRKY46-*overexpressing transgenic *Arabidopsis* plants (Figure 8). It suggested that TaWRKY46 appeared to be a regulator involved both in ABA-dependent and ABA-independent pathways. Similar findings were also found in the functional characterizations of TaWRKY2, TaWRKY19, TaWRKY93, and TaMYB73 transcription factors [21,22,47]. It was partly because that a single WRKY TF can be involved in regulating several seemingly disparate processes [30,44]. These results were in accordance with previous findings that plants promoted their responses to abiotic stresses by enhancing the expression of stress-related genes [48,49,50,51,52,53]. Therefore, inducing stress-related genes may be the dominant mechanism to enhancing osmotic stress tolerance. If TaWRKY46 could directly bind to the upstream region of these genes is an idea that needs to be further explored, which will illuminate the underlying molecular mechanism of TaWRKY46 involved in the osmotic stress signal pathway in wheat.

## 4. Materials and Methods

### 4.1. Hydroponic Wheat and Stress Treatments

Winter wheat variety Zhengyin 1 (drought-sensitive) was utilized in the experiment. The seeds were sterilized in 75% ethanol (*v*/*v*) for 10 min, rinsed six times with distilled water, and germinated in a glass dish with wet filter paper (Zhejiang, China) for 36 h in the dark. The germinated seeds were transferred into pots filled with 1/2 Hoagland solution (Qingdao, China, Supplementary Figure S2A). The pots were cultured in a growth chamber with a day/night temperature of 25 °C/20 °C and 16 h/8 h photoperiod at 180 µmol m^−2^ s^−1^ illumination. The 1/2 Hoagland solution was refreshed every 2 days. When the second leaf fully expanded, 8-day-old seedlings (Supplementary Figure S2B) were treated with 1/2 Hoagland solution containing 20% PEG 6000, 200 mM NaCl, and 100 μΜ ABA for 24 h under the same chamber. Low- and high-temperature treatments were conducted at 4 °C and 42 °C chambers for 24 h, respectively. Seedlings cultivated in free 1/2 Hoagland solution were taken as a control. For the multiple stress experiments, the leaves were sampled at 0, 1, 3, 6, 12, and 24 h after PEG, NaCl, ABA, and low- and high-temperature treatments. For the organ expression analysis, the leaf, root, and stem were collected from control seedlings at 0 h. All the samples were immersed immediately into liquid nitrogen and stored at −80 °C for RNA extraction. The specific gene primers were listed in Supplementary Table S2.

### 4.2. Gene Isolation and Bioinformatics Analysis of TaWRKY46

The complete coding sequence of *TaWRKY46* was amplified by PCR using specific primers (Supplementary Table S2). The PCR product was ligated to the *pMD19-T* vector (TaKaRa, Dalian, China) and then sequenced by the company (Sangon Biotech, Shanghai, China). The exon–intron structure of *TaWRKY46* was obtained by the Gene Structure Display Server 2.0. TaWRKY46 and its highest homologies in other species including monocotyledonous grasses (barley, rice, and maize), and dicotyledonous soybean and Arabidopsis, were selected to generate a phylogenetic tree. The tree was constructed by MEGA 6 software using the Neighbor-Joining (NJ) method with 1000 bootstrap replicates. The accession numbers of the *WRKYs* were listed in Supplementary Table S1. The tertiary structure of TaWRKY46 protein was acquired through the Phyre2 tool (http://www.sbg.bio.ic.ac.uk/phyre2) and further edited by Chimera 1.11.2 software. Multiple sequence alignment was performed by DANMAN software (Lynnon Biosoft, San Ramon, California, USA).

### 4.3. Subcellular Localization Analysis of TaWRKY46

The cDNA encoding full-length *TaWRKY46* without the stop codon was inserted into the *pTF486-GFP* vector [54] after it was cloned by PCR using specific primers (Supplementary Table S2) with *Bam*HI restriction site. Wheat leaf protoplasts were isolated from 10-day-old seedlings according to the method of Shan [55]. *pTF486-TaWRKY46-GFP* was transformed into protoplasts by the PEG-mediated method and then incubated at 22 °C for 12 h in the dark. Empty vector was used as a control. GFP fluorescence signals were visualized using a laser confocal microscope (Andor, Belfast, UK) at an excitation wavelength of 488 nm.

### 4.4. Analysis of Transcriptional Activation in Yeast

The TaWRKY46 protein had 294 amino acid residues. The 1-191 amino acid residues contained the completed WRKY domain and C_2_HC zinc finger motif. The 191^th^ amino acid position was chosen for truncation. The full-length coding sequence and truncated segments of *TaWRKY46* were fused to the *pGBKT7* vector [56] with *Eco*RI and *Bam*HI restriction sites. The specific primers were listed in Supplementary Table S2. The various constructions were named *pGBKT7-TaWRKY46* (1-294 aa), *pGBKT7-TaWRKY46* (1-191 aa), and *pGBKT7-TaWRKY46* (192-294 aa), respectively. Empty vector *pGBKT7* was taken as a control. These constructions were introduced into the yeast strain *AH109* by the lithium acetate method. The yeast cells were grown on selective medium (SD) medium without tryptophan (SD-W) and screened by PCR. The positive clones were cultured on the SD-W, SD without tryptophan, histidine, and adenine (SD-W/H/A) and SD-W/H/A with X-α-D-Galactosidase (X-α-gal) medium at 30 °C. The images were photographed after 3 d to detect the transcriptional activation.

### 4.5. Plant Transformation and Generation of TaWRKY46-Overexpressing Arabidopsis Plants

The complete coding sequence of *TaWRKY46* was ligated into a binary vector *pBI1304* [57] with *Nco*I and *Spe*I restriction sites under control of *Cauliflower mosaic virus* 35S (CaMV35S) promoter using the specific primers (Supplementary Table S2). The construction was introduced into *Agrobacterium tumefaciens* strain *GV3101* by the freeze–thaw method. The floral dip method was used for generating transgenic *Arabidopsis* lines [58]. T_1_ plants were screened on 1/2 MS medium containing 1% sucrose, 1% agar, and 25 μg ml^−1^ hygromycin. T_1_ seedlings were confirmed by PCR and selfed through two more generations to generate T_3_ transgenic progeny. Sixteen independent transgenic T_3_ lines were acquired, and the transcriptional level of *TaWRKY46* in the two selected overexpression (OE) lines was validated by PCR and qRT-PCR.

### 4.6. Water Loss Rate and Survive Rate Assays

Wild-type (WT) and *TaWRKY46*-overexpressing *Arabidopsis* seeds were sterilized in 10% (v/v) sodium hypochlorite (NaClO) for 10 min, rinsed six times with sterilized distilled water and vernalized at 4 °C for 3 days. *Arabidopsis* was grown on Jiffy-7-Peat Pellets (Jiffy Group) and cultivated in a growth chamber kept at 22 °C at 80 µmol m^−2^ s^−1^ illumination in a 16 h/8 h day/night cycle. For water loss rate assay, the detached leaves from 24-day-old WT and *TaWRKY46*-overexpressing *Arabidopsis* T_3_ seedlings were placed on filter papers at room temperature for 0, 1, 2, 3, 4, 5, 6, and 12 h, and the mass of the leaves was weighted at the indicated interval. Three replicates were performed. The 24-day-old seedlings were subjected to water withholding for 25 days, and 60 seedlings from each line were used to determine the survival rate.

### 4.7. Seed Germination Rate Assay

For the seed germination rate assay, WT and *TaWRKY46*-overexpressing transgenic *Arabidopsis* T_3_ seeds were sterilized and grown on 1/2 MS medium supplemented with 0 mM, 150 mM, and 300 mM mannitol for 4 days. The seed germination rate was recorded every day. At least 50 seeds were sown for one replicate of three parallel replicates. Three independent experiments of germination rate assay were performed. Measurements were repeated three times.

### 4.8. Stress Tolerance Assay and Expression Analysis of Stress-Related Genes in TaWRKY46-Overexpressing Arabidopsis Plants

WT and *TaWRKY46*-overexpressing *Arabidopsis* seeds were surface sterilized and planted on 1/2 MS medium containing 1% sucrose and 1% agar after 3 days of stratification at 4 °C. The plates were cultured vertically in a growth chamber at 22 °C under continuous illumination at 80 µmol m^−2^ s^−1^. For the salt stress assay, 5-day-old seedlings were transferred to 1/2 MS medium supplemented with 0, 100 and 200 mM NaCl. For the osmotic stress assay, 5-day-old seedlings were transferred to 1/2 MS medium supplemented with 0 mM, 150 mM, and 300 mM mannitol. The root lengths were measured using the software ImageJ after 10 days treatment. 15-day-old WT and transgenic *Arabidopsis* seedlings were collected from the 1/2 MS medium under 0, 150 and 300 mM mannitol treatments for further gene expression analysis. The expression of stress-related genes was detected by qRT-PCR. The specific primers were listed in Supplementary Table S2**.** Measurements were repeated three times.

### 4.9. RNA Extraction and qRT-PCR Analysis

Total RNA was isolated using the Trizol extraction protocol (Invitrogen, Carlsbad, California, USA) following the manufacturer’s instructions. Complementary DNA (cDNA) was synthesized from 1 μg total RNA by the PrimeScript™ II 1st Strand cDNA Synthesis Kit (TaKaRa, Dalian, China), and the quantitative real-time PCR (qRT-PCR) was carried out using SYBR^®^ Premix Ex Taq™ II (TaKaRa, Dalian, China) on a CFX96 Touch RealTime PCR Detection System (BioRad, Hercules, California, USA). The specific gene primers were designed by Primer Premier 6.0 software and listed in Supplementary Table S2. The relative gene expression was calculated through the formula 2^-ΔΔCT^ method [59]. Three biological and three technical replicates were performed in gene expression analysis. Three independent experiments were performed.

### 4.10. Statistical Analysis

Microsoft Office Excel 2013 was first used to analyze the data, and the mean values were calculated from three parallel experiments with their standard errors. Duncan’s method was performed using the SPSS Statistics 20.0 software. GraphPad Prism 7, Adobe Photoshop CC, and Adobe Illustrator software were used to generate charts.

## 5. Conclusions

A wheat Group III gene, *TaWRKY46*, was up-regulated by PEG treatment. Overexpression of *TaWRKY46* activated the stress-related genes, increased the germination rate, and promoted the root growth in transgenic *Arabidopsis* plants under osmotic stress. These findings indicated that TaWRKY46 was a new positive regulator of osmotic stress. It played an important role in improving osmotic tolerance through ABA-dependent and ABA-independent pathways when plants were exposed to osmotic stress.

## Supplementary Materials

The following are available online at https://www.mdpi.com/1422-0067/21/4/1321/s1, Supplementary Materials consist of two tables and two figures. Supplementary Table S1: The accession numbers of the *WRKYs* used to construct phylogenetic tree in Figure 1. Supplementary Table S2: List of primers used in this study. F and R represent the forward and reverse primers from 5’ end to 3’ end. Supplementary Figure S1: Salt stress assay of *TaWRKY46*-overexpressing *Arabidopsis* plants. (A-C) The root length statistics of seedlings after treatments with 0 mM,100 mM, and 200 mM NaCl for 10 days. Lowercase letters represent significance at *p <* 0.05 between WT and transgenic Arabidopsis lines under the same condition, as determined by Duncan’s multiple range test. Supplementary Figure S2: The growth status of wheat seedlings. The overview of hydroponic wheat in plastic pots filled with 1/2 Hoagland solution (A) and the phenotype of 8-day-old seedlings before various treatments (B).

## Abbreviations

ABF	ABA-response element-binding factor
CBF	C repeat/dehydration-responsive element-binding factor
GFP	green fluorescent protein
OE	overexpression
P5CS	Δ-1-pyrroline-5-carboxylate synthetase
RD	dehydration-responsive
TFs	transcription factors
WT	wild type
