# Supplementary material for: A Wheat WRKY Transcription Factor TaWRKY46 Enhances Tolerance to Osmotic Stress in transgenic Arabidopsis Plants
Source: Int J Mol Sci. 2020 Feb 15;21(4):1321. doi: 10.3390/ijms21041321 (PMC7072902; doi:10.3390/ijms21041321)
Supplement: Supplementary file 1 [file ijms-21-01321-s001.zip › Supplementary Files/Supplementary Tables S1-S2.docx]

**Supplementary Table S1.** The accession numbers of the *WRKYs* used to construct phylogenetic tree in **Figure 1**.

| Genes | Accession No. |
| --- | --- |
| *TaWRKY46* | TRAES3BF051200110CFD_t1 |
| *Hv1065C13* | AK357930.1 |
| *OsWRKY53* | XM_015776833.2 |
| *ZmWRKY24* | XM_008677041.2 |
| *AtWRKY30* | AF404858.1 |
| *AtWRKY41* | NM_117177.3 |
| *AtWRKY53* | NM_118512.3 |
| *GmWRKY41* | XM_003525301.4 |

**Supplementary Table S2.** List of primers used in this study. F and R represent the forward and reverse primers from 5' end to 3' end.

| **Purpose** | **Name** | **Primers (5' - 3')** |
| --- | --- | --- |
| qRT-PCR | *TaWRKY46-F* | CACGGCGACCCGCTGCTCTT |
|  | *TaWRKY46-R* | GCTGTGGCCTCTGGCTGTTG |
|  | *TaTubulin-F* | ACCGTGGTGATGTTGTGC |
|  | *TaTubulin-R* | CCTGGTGGCTGGTAGTTGA |
| Clone-PCR | *TaWRKY46-F* | ATGGAGGAGAGGTGCGCCC |
|  | *TaWRKY46-R* | TTAGAGATAGCGCGAGTTATCGA |
| Subcellular  location | *pTF486-TaWRKY46- GFP-F* | CGGGATCCATGGAGGAGAGGTGCGCC |
|  | *pTF486-TaWRKY46- GFP-R* | CGGGATCCGAGATAGCGCGAGTTATCGAGG |
| Transcriptionalactivation | *pGBKT7-TaWRKY46(1-294 aa)-F* | GCCATGGAGGCCGAATTCATGGAGGAGAGGTGCGCC |
|  | *pGBKT7-TaWRKY46(1-294 aa)-R* | CCGCTGCAGGTCGACGGATCCTTAGAGATAGCGCGAGTTATCGAG |
|  | *pGBKT7-TaWRKY46(1-191 aa)-F* | GCCATGGAGGCCGAATTCATGGAGGAGAGGTGCGCC |
|  | *pGBKT7-TaWRKY46(1-191 aa)-R* | CCGCTGCAGGTCGACGGATCCCTGGCTGTTGTAGTGCTTGCC |
|  | *pGBKT7-TaWRKY46(192-294 aa)-F* | GCCATGGAGGCCGAATTCAGGCCACAGCCCGTAGCA |
|  | *pGBKT7-TaWRKY46(192-294 aa)-R* | CCGCTGCAGGTCGACGGATCCTTAGAGATAGCGCGAGTTATCGAG |
| Over-expression | *pBI1304-TaWRKY46-F* | GGACTCTTGACCATGGAGGAGAGGTGCGCCC |
|  | *pBI1304-TaWRKY46-R* | CTTCTCCTTTACTAGTTTAGAGATAGCGCGAGTTATCGA |
| qRT-PCR | *AtTubulin-F* | AAGGGACACTACACGGAAGGA |
|  | *AtTubulin-R* | GGAACACCGAGAAGGTAAGCA |
|  | *AtP5CS1-F* | CTTGTGATACGGATATGGCAAAGCG |
|  | *AtP5CS1-R* | CCTTGGTCCACCATACAAAGTGACTCC |
|  | *AtRD29B-F* | AGAAGGAATGGTGGGGAAAG |
|  | *AtRD29B-R* | CAACTCACTTCCACCGGAAT |
|  | *AtDREB2A-F* | TGACCTAAATGGCGACGATGT |
|  | *AtDREB2A-R* | TCCAAGTAACTCAAGTCGTCG |
|  | *AtDREB1C/CBF2-F* | AACCAGCGGGAAGGAAGAAGT |
|  | *AtDREB1C/CBF2-R* | TTTCCTTGGCACAGGTTGATT |
|  | *AtDREB1A/CBF3-F* | GATCAGCCTGTCTCAATTTC |
|  | *AtDREB1A/CBF3-R* | CTTCTGCCATATTAGCCAAC |
