# Supplementary figures and images for: A Wheat WRKY Transcription Factor TaWRKY46 Enhances Tolerance to Osmotic Stress in transgenic Arabidopsis Plants
Source: Int J Mol Sci. 2020 Feb 15;21(4):1321. doi: 10.3390/ijms21041321 (PMC7072902; doi:10.3390/ijms21041321)

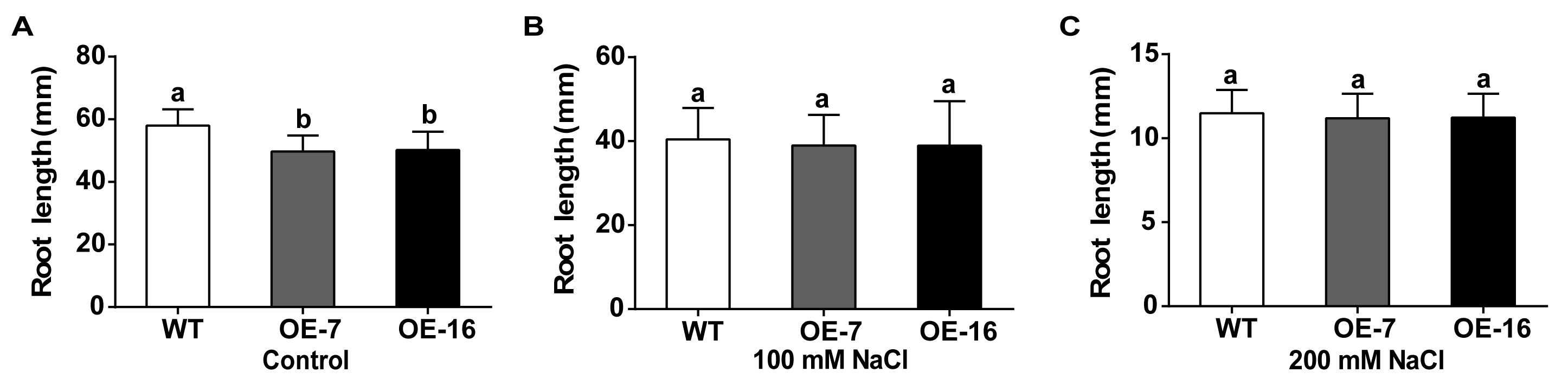

Supplement: Supplementary file 1 [file ijms-21-01321-s001.zip › Supplementary Files/Supplementary Figure 1.tif]

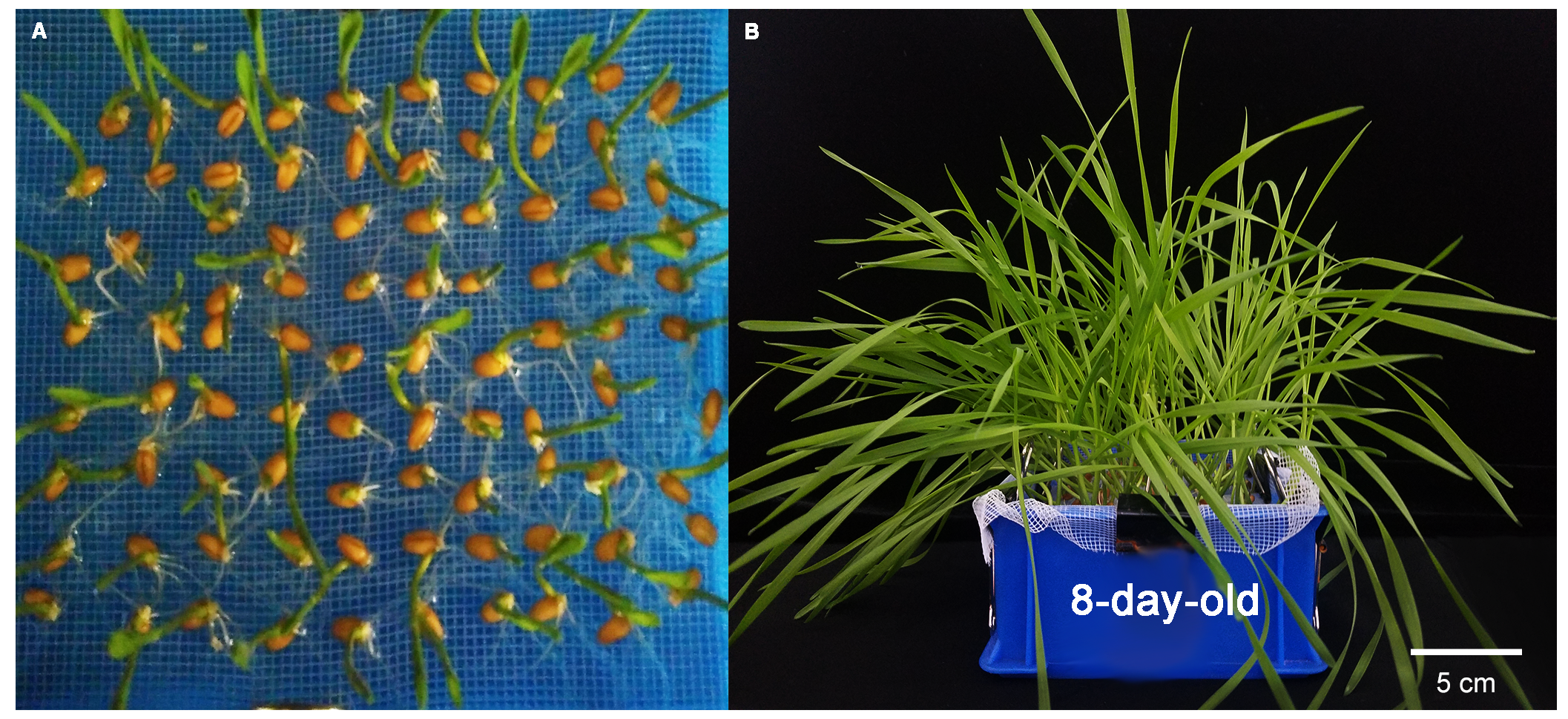

Supplement: Supplementary file 1 [file ijms-21-01321-s001.zip › Supplementary Files/Supplementary Figure 2.tif]
